# Organisational systems’ approaches to improving cultural competence in healthcare: a systematic scoping review of the literature

**DOI:** 10.1186/s12939-017-0571-5

**Published:** 2017-05-12

**Authors:** Janya McCalman, Crystal Jongen, Roxanne Bainbridge

**Affiliations:** 1 School of Health, Medicine and Applied Sciences, Central Queensland University, Cnr Shields and Abbott Streets, Cairns, 4870 QLD Australia; 2 Centre for Indigenous Health Equity Research, Central Queensland University, Cnr Shields and Abbott Streets, Cairns, 4870 QLD Australia

**Keywords:** Cultural competency, Indigenous, Ethnic minorities, Health disparities, Health systems, Health services

## Abstract

**Introduction:**

Healthcare organisations serve clients from diverse Indigenous and other ethnic and racial groups on a daily basis, and require appropriate client-centred systems and services for provision of optimal healthcare. Despite advocacy for systems-level approaches to cultural competence, the primary focus in the literature remains on competency strategies aimed at health promotion initiatives, workforce development and student education. This paper aims to bridge the gap in available evidence about systems approaches to cultural competence by systematically mapping key concepts, types of evidence, and gaps in research.

**Methods:**

A literature search was completed as part of a larger systematic search of evaluations and measures of cultural competence interventions in health care in Canada, the United States, Australia and New Zealand. Seventeen peer-reviewed databases, 13 websites and clearinghouses, and 11 literature reviews were searched from 2002 to 2015. Overall, 109 studies were found, with 15 evaluating systems-level interventions or describing measurements. Thematic analysis was used to identify key implementation principles, intervention strategies and outcomes reported.

**Results:**

Twelve intervention and three measurement studies met our inclusion criteria. Key principles for implementing systems approaches were: user engagement, organisational readiness, and delivery across multiple sites. Two key types of intervention strategies to embed cultural competence within health systems were: audit and quality improvement approaches and service-level policies or strategies. Outcomes were found for organisational systems, the client/practitioner encounter, health, and at national policy level.

**Discussion and implications:**

We could not determine the overall effectiveness of systems-level interventions to reform health systems because interventions were context-specific, there were too few comparative studies and studies did not use the same outcome measures. However, examined together, the intervention and measurement principles, strategies and outcomes provide a preliminary framework for implementation and evaluation of systems-level interventions to improve cultural competence. Identified gaps in the literature included a need for cost and effectiveness studies of systems approaches and explication of the effects of cultural competence on client experience. Further research is needed to explore the extent to which cultural competence improves health outcomes and reduces ethnic and racially-based healthcare disparities.

## Introduction

Healthcare organisations serve clients from diverse Indigenous and other ethnic and racial groups on a daily basis, and require appropriate client-centred systems and services in order to provide optimal healthcare. Yet there is extensive research evidence demonstrating that racial and ethnic minorities do not receive equal treatment when accessing healthcare services [1]. Disparities can result from discriminatory treatment by healthcare practitioners [2], and can also be ameliorated by the actions of healthcare organisations. Cultural competence has been identified as one strategy to address racial and ethnic health disparities in healthcare by providing services that meet clients’ cultural, social, and communication needs [3–5].

The concept of cultural competence was first identified in the late 1980s to address the effects of cultural and linguistic barriers in the interpersonal encounter between healthcare practitioners and clients on health service access and delivery [6]. It was considered that individual health practitioners needed to be capable of functioning effectively in cross- cultural contexts [7], and, that this required them to develop awareness of cultural differences [6]. Recognising the important role of organisations, the scope of cultural competence expanded beyond the interpersonal domain of cross-cultural care to address multiple levels including health systems [6].

Cultural competence was defined in the late 1980s as a “set of congruent behaviours, attitudes and policies that come together in a healthcare system, agency or among professionals that enable that system, agency or professions to work effectively in cross- cultural situations” [8]. The responsibility for cultural competence was therefore considered not just to concentrate in single healthcare services, but to also require broader system- wide policies [9]. It was argued that a systems approach to cultural competence is required, because: “The bottom line is that clinicians and caregivers cannot on their own drive and follow practices that lead to culturally and linguistically appropriate care” [2]. The earlier broad definition of cultural competence by Cross, Bazron [8] was reiterated in 2008 by the United States (US) National Quality Forum [10] as the “ongoing capacity of healthcare systems, organisations and professions to provide for diverse client populations high quality care that is safe, client and family-centred, evidence-based and equitable”.

Systems approaches are increasingly being applied in the delivery and management of various aspects of healthcare [11]. A systems perspective considers healthcare organisations as systems comprised of interrelated and interdependent components: client care; ancillary services; professional staff; and financial, informational, physical and administrative subsystems [12, 13]. Systems thinking focusses attention on how components are connected to each other within a whole entity, how components work together to achieve an intended outcome, and thereby how systems can be changed to produce better outcomes [11]. A systems approach to cultural competency integrates practices throughout the organisation’s management and clinical sub-systems, thus requiring an amalgamation of attitudes, practices, policies and structures to enable healthcare organisations and professionals to work effectively in culturally diverse situations [8]. An organisation becomes more culturally competent by adapting these systems and subsystems to the needs of its diverse workforce and client population [13].

In the decades since the first definition of cultural competence, racial and ethnic diversity has increased in Canada, Australia, New Zealand (NZ) and the US (the CANZUS nations), with Census projections predicting continuing diversification [2]. In these nations, Indigenous and other ethnic and minority peoples, particularly those with limited English proficiency, share poorer health and life expectancies than the majority populations [1, 3, 14–16]. Hence, the mandate for systems-level cultural and linguistic competence to reduce disparities in healthcare has strengthened.

Multi-levelled and multi-strategic systemic responses have been enacted in the four countries to improve cultural competence. At national levels, the governments of NZ and the US have enacted legislation (NZ Health and Disability Act and US National Standards for Culturally and Linguistically Appropriate Services (CLAS) in health and health care which have been legislatively mandated by at least six states) to improve culturally competent care, language access services and organisational supports for cultural competence [13, 17–19]. The US National Quality Framework reiterated its commitment by identifying six domains for cultural competency: 1) leadership; 2) integration into management systems and operations; 3) workforce diversity and training; 4) community engagement; 5) client- provider communication; and 6) care delivery and support mechanisms [10]. Australia has recently renewed its national framework for cultural respect [20] and in Canada, the broad *Multiculturalism Act* is aimed at providing all citizens with equal access and opportunities to ensure that needs associated with culture are considered in decision-making processes [21]. National professional associations have also developed healthcare practitioner competency standards.

At regional and local levels, healthcare organisations, including hospitals and primary healthcare services are increasingly recognising cultural competence as an organisational strategy to address the needs of diverse client populations [4]. Healthcare organisations have developed policies; workforce education and training programs; audit, monitoring and quality improvement practices; and culturally tailored programs and services [6, 15, 22–28]. Despite some healthcare organisations being responsive to the cultural and linguistic needs of their client populations, the required financial investments and failure to recognise the potential benefits mean that some organisations do not implement cultural competence interventions [13].

Despite such efforts to enact systems-level approaches to cultural competence, few studies have described or assessed the extent of systems approaches to cultural competence [13]. This paper aims to bridge the gap in available evidence about systems approaches to cultural competence by systematically searching, selecting and synthesising existing publications to map key concepts, types of evidence, and gaps in research [29]. By so doing, the review will provide healthcare organisations with guidance to implement systematic approaches to cultural competence by identifying the mix of strategies that work in practice, principles for implementing them, and the extent to which they can expect improvements in clients’ experiences of healthcare and their health outcomes. As suggested by Dijkers [30], assessments of the quality of studies are included to provide confidence that the implications of the review for policy, practice or clients, are based on high quality research. The research question was: What is the current evidence base for the impact of systems level approaches to cultural competence?

The objectives of the paper are to:

1. Identify systems-level interventions that have been evaluated in the literature;
2. Report the effects of these interventions in improving cultural competence;
3. Report on how cultural competence at a systems-level has been measured;
4. Summarise the quality of available evidence.

## Method

The paper is based on the results of a broader systematic scoping review of the literature to identify intervention strategies and indicators which have been applied to increase cultural competency in health care, along with the outcomes of these interventions [31]. The review of cultural competence in health care in Australia, NZ, Canada and the US was undertaken first in July 2012 and updated in June 2016. The four CANZUS nations were selected because they share a history of settler colonisation by Britain, similar legacies of English common law, political governance, language, settlement and culture, and health systems [32].

However, important contextual differences in broad national healthcare systems and cultural competence approaches affect its implementation.

### Search strategy

The search strategy employed for the review comprised an initial search in 2012, for the period 1 January 2002–31 July 2012. The start date was determined by the seminal US Institute of Medicine report on Unequal Treatment: Confronting Racial and Ethnic disparities in healthcare [1] which highlighted systemic disparities in health care and health status for racial and ethnic minority populations. The search was updated in June 2016 for the period 1 August 2012–31 December 2015.

**Fig. 1:**
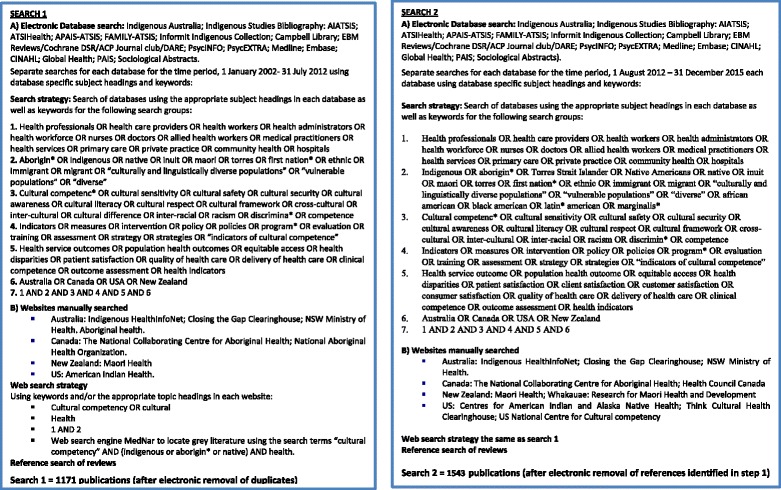
Search strategies

For each search, a qualified librarian systematically searched 17 electronic databases and relevant websites (Fig. 1). Peer-reviewed and grey literature (including government and agency reports) published in English were included. The references of reviews of cultural competency in healthcare were hand-searched for additional relevant studies.

### Inclusion criteria

Studies were included in the broader review [31] if they:

1. Explicitly focussed on cultural competence in relation to Indigenous and other minority ethnic and racial groups in Australia, Canada, NZ or the US. That is, studies aimed to improve cultural competence or included an indicator of cultural competence (or like terms). Included studies were designed to addresscultural awareness of health staff; Indigenous or ethnic minority peoples’ access to health services, procedures, and/or culturally specific programs; the provision of culturally respectful services; Indigenous or ethnic minority workforce development; and culturally tailored interventions. We did not include studies with a primary focus on racial or ethnic disparities in health, the recruitment and retention of staff members who reflect the cultural diversity of the community served, nor the identification of Indigenous peoples or ethnic minorities in health service records.
2. Related to cultural competence in any health care service (i.e. hospitals, primary health care settings, specialist health areas, private practice and community health settings), and for both health care outcomes and population health outcomes;
3. Were intervention evaluations or studies of indicators/measures of cultural competency. Following Sanson-Fisher, Campbell [33], intervention evaluations were defined as studies that evaluated the effectiveness of a strategy, service, program or policy designed to improve cultural competency. Indicator/measurement studies were defined as studies that described, developed, tested or applied measures/indicators of cultural competence.

### Identification, screening and inclusion of publications

**Fig. 2:**
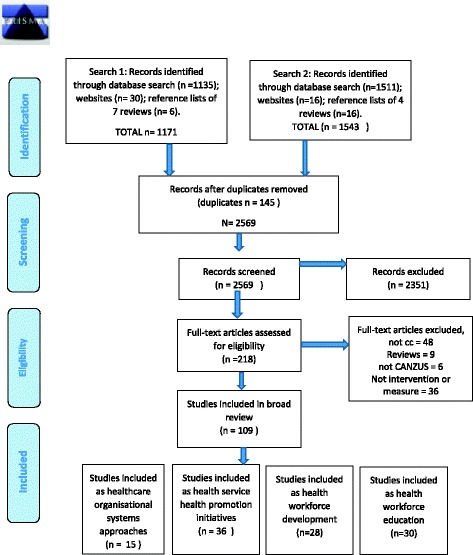
PRISMA flow diagram

As shown in the Preferred Reporting Items for Systematic Reviews and Meta-Analyses (PRISMA) diagram [34] in Fig. 2, a total of 1171 publications were identified in the first search and a further 1543 publications in the second search. The titles and abstracts of these 2714 publications were imported into the bibliographic citation management software, EndNote X7, duplicates removed and their abstracts manually examined to identify evaluations of strategies to improve cultural competency in health care or indicators of cultural competency. One author (RB) screened the first search and a second author (CJ) retrieved and screened titles and abstracts of the remaining publications from the second search; those which did not meet inclusion criteria were excluded. The full texts of the remaining publications were retrieved and screened by blinded reviewers (RB, JM). Inconsistencies in reviewer assessments were resolved by consensus.

In this paper, we report on the evidence for healthcare systems approaches to cultural competence. A total of 141 publications relevant to this review were included in the broader review. For the purposes of this review, studies focussed on healthcare organisational systems approaches were mined and extracted from the broader review.

### Data analysis

Data that related to the author, year and type of publication; country where developed and population; health care setting; type of intervention/measurement; healthcare outcomes assessed; outcome indicator and/or measure; study design and study quality were extracted (Table 1). The quality of intervention studies was assessed using the Effective Public Health Practice Project (EPHPP) quality assessment tool for quantitative studies and Critical Appraisal Skills Programme (CASP) quality assessment tool for qualitative studies.

**Table 1:** Publications which evaluated or provided measures for systems-level cultural competence interventions

Author, year & publication	Country and population	Health care setting	Type of intervention	Healthcare outcomes	Outcome indicator and/or measure	Study design	Study quality
Chong, Renhard [36] Paper	Australia Aboriginal and Torres Strait Islander clients	Hospitals	The Improving the Culture of Hospitals Project developed and trialled an evidence based quality improvement ‘toolkit’ to support continuous quality improvement (CQI) for improving cultural sensitivity. Aboriginal staff were trained in the use of CQI technology. A national key stakeholder forum to explore research and implementation.	Hospitals that have improved cultural sensitivity share: relationships with Aboriginal communities and commitment to supporting the Aboriginal workforce.	Cultural sensitivity	Qualitative: Case studies—continuous quality improvement.	Moderate
Freeman, Edwards [37] Paper	Australia Aboriginal and Torres Strait Islander Australians	An Aboriginal community controlled health care service and a state government-managed primary healthcare service.	Appraisals of the achievement of cultural respect following health- service-level strategies for culturally respectful care.	Implementation enablers: being grounded in a social view of health, advocacy and addressing social determinants; employing Aboriginal staff; creating a welcoming service; supporting access through transport, outreach, and walk-in centres; and integrating cultural protocol. Barriers: communication difficulties; racism and discrimination; and externally developedprograms. Service-level strategies are necessary for achieving cultural respect.	Staff and clients reported on cultural respect strategies, client experiences and barriers to cultural respect.	Two case studies—22 interviews with staff, an audit and survey, four community assessment workshops with 21 clients.	Weak
Liaw, Hasan [38] Paper	Australia Aboriginal people	General practice and primary care organisations.	A cultural respect workshop provided orientation to the ‘*Ways of Thinking Ways of Doing’* clinical re-design program to improve the cultural competency of General Practices. Support from a cultural mentor and a toolkit to guide activities to embed cultural respect into practice.	Identification of Aboriginality of new and existing clients, practice organisational arrangement, chronic disease risk factor recorded, health assessment billed, practitioners’ cultural quotient (cultural strategic thinking, motivation, behaviour).	A generic cultural quotient questionnaire and audit of Aboriginal clients identified, health checks done and clinical risk factors managed.	A pragmatic pre- and post- study using qualitative interviews and quantitative audit and survey.	Weak
Lieu, Finkelstein [43] Paper	US Non-English speakers and clients with low literacy	Primary health care for Medicaidinsured children with asthma	Aimed to identify practice-site policies and features associated with quality of care for Medicaidinsured children with asthma.	At one-year follow up, clients of practice sites with the highest cultural competence scores were less likely to be underusing preventive asthma medications and had better parent ratings of care.	Health care cultural competence policies and procedures, client selfmanagement, empowerment and communication.	Telephone interviews with parents, surveys of practice sites and computerised databases.	Strong
Noe, Kaufman et al. [42] Paper	US American Indian and Alaska Native (AI/AN) veterans	Access to appropriate care for AI/AN veterans	What organisational characteristics predict the provision of culturally competent services.	Only 15% services reported that their facilities provided traditional healing services. Mean scores were above the midrange on all organisational readiness to change measures.	Organisational readiness to change. Included items relating to AI/AN veterans’ services and projects.	Adapted Organisational Readiness to Change Assessment survey (needs, leadership, resources, and organisational climate scales).	Weak
O'Brien, Boddy [39] Paper	NZ (but includes Australian standards) Maori and non- Maori mental health clients	Mental health services and mental health nursing	Health service audit measure using bicultural indicators for clinical records and cultural competence.	Wide variation across services, especially in informed consent, information about legal rights, and culturally safe and recovery-focussed care.	Health service Consumer Notes Clinical Indicators (CNCI) audit tool.	Four phased design: 1) focus groups with expert mental health nurses; 2) Delphi surveys; 3) a pilot study; 4) national audit of mental health services.	Moderate
O'Brien, Boddy [40] Paper	NZ and Australia Indigenous peoples	Mental health care	Health service audit measure using bicultural indicators for clinical records and cultural competence -Ascertaining the degree to which quality improvement and monitoring systems are enhancing professional practice and client outcomes.	Variation in cultural competence of nursing practice across mental health services. The way in which services were delivered impacted upon clients’ ability to engage in the treatment processes and ultimately in their recovery; clients became more involved in their own care; kin and community became more involved in care. Indicators identified areas of clinical nursing care needing improvements.	Clinical indicators of critical events - Consumer Notes Clinical Indicators (CNCI) audit tool	Audit of mental health services	Moderate
Reibel and Walker [41] Paper	Australia Aboriginal women	Ante natal services in WA	Client access or utilisation of health service by racial or ethnic group.	Only 9/42 audited services which reported utilisation by Aboriginal women had achieved a model of culturally responsive service delivery (i.e. incorporated Aboriginal specific antenatal protocols/programs, maintained access, employed Aboriginal Health Workers). The indicators established benchmarks for planning culturally appropriate antenatal services.	Access and quality of care of health services (general characteristics, risk assessment, treatment risk reduction and education, access and quality of care); indicators of cultural responsiveness.	Audit. Purposespecific audit tool administered through telephone interviews.	Weak
Weech-Maldonado, Elliot [5] Paper	Ethnic/racial minority clients	Hospitals	Aimed to assess whether greater cultural competence in hospitals improves client experiences, particularly for ethnic/racial minority clients.	Greater cultural competence was positively associated with doctor communication, overall hospital rating and hospital recommendation. There were greater relative benefits for non-Englishspeaking non-Hispanic whites.	Client experience with care (communication with doctors and nurses, staff responsiveness, pain control, communication about medications, discharge information, cleanliness of hospital, quietness of hospital, recommendations of hospital to friends and family, overall rating) with hospital cultural competence and selfreported client race, ethnicity and language.	Exploratory single timepoint correlation between national Consumer Assessment of Healthcare Providers and Systems (CAHPS) hospital survey scores and Cultural Competence Assessment Tool for Hospitals (CCATH) scores.	Moderate
Whelan, Weech-Maldonado [44] Paper	US and Australia Diversity management by Senior	Hospitals	Comparative evaluation of how diversity management is enacted in hospitals across two countries.	Both Australian and US hospitals can do much more to implement best practices in diversity management. Australian hospitals scored higher on organisational change indicators; US hospitals on human resource indicators but there was more similarity than difference. Despite 30–40 years of “multicultural health”, neither has achieved best practice.	Diversity management activities (planning, stakeholder satisfaction, diversity training, human resources, health care delivery, organisational change, diversity performance, external and internal influences on racial/ethnic diversity initiatives).	Comparative exploratory study based on single point in time surveys.	Weak
Whitman and Davis [45] Paper	US Diversifying client population	Cross- 101 general medical and surgical hospitals in Alabama sample of 53 respondents.	Examined the awareness of and preparedness for the diversifying client population through their hospital cultural and linguistic competence practices.	Hospitals are taking initial steps to meet the needs of the diversifying population, but have a long way to go to meet National Standards for culturally and linguistically appropriate services in health care.	Hospital adherence to national standards.	Self-report questionnaires to hospital chief executive officers on the measures and resources that the hospitals currently use to meet cultural and linguistic	Weak
Wiley [18] Paper	NZ Maori who are disabled	Knowledge and attitudes to cross-cultural disability care.	Consumers, carers, service providers and policy makers’ knowledge and attitudes.	Conflict between Indigenous worldviews framed within a mainstream service; Need for increased coordination and collaboration, workforce development, resources and information development, and community engagement.	Disability care and participation in services.	Semi-structured interview instrument, focus groups.	Weak
O'Brien, O'Brien [46] Paper	NZ Maori and non-Maori mental health clients	Mental health services and mental health nursing.	Health service audit tool using bicultural indicators for clinical records and cultural competence.	No health care outcomes reported (paper describes development of indicators by expert committee).	Health care delivery	Development of Consumer Notes Clinical Indicators (CNCI) for clinical records and cultural competence and Professional Practice Audit Questionnaire (PPAQ) self-report survey.	
Siegel, Haugland [47] Paper	US African American, Hispanic, Asian and American Indian	Mental health services	Audit tool to measure the cultural competence of health services.	No health care outcomes reported (paper describes development by expert committee).	Health care delivery	Development of health service benchmarking audit tool and self-report survey.	
Weech-Maldonado, Dreachslin [48] Paper	US Hospitals	Hospitals in California	Pilot tested an initial draft of the Cultural competency assessment tool for hospitals (CCATH), revised then field tested it with a sample of hospitals.	The CCATH can be used to evaluate hospital performance in cultural competency and identify improvements. Not for profit hospitals had higher CCATH scores than for profit.	The CCATH scales were reliable.	Development of Cultural Competency Assessment Tool of Hospitals (CCATH).	

Thematic analysis methods [35] were used to identify key themes across evaluations. A mind map was constructed to sort the intervention strategies utilised and their associated outcomes. Overarching themes were then reviewed, refined and named [35].

## Results

We found 15/109 (13.8%) papers that met the inclusion criteria as evaluating or providing measures for systems approaches to cultural competence. Of these, 12 were intervention studies and three were measurement studies. There was a significant variation in focus, content, mode of delivery and duration of interventions. There was also heterogeneity in the outcomes reported across the studies. A summary of the extracted characteristics of the included studies is provided in Table 1. Measurement studies are shaded.

### Publication year

The quantity of publications was somewhat evenly spread over the time period (2002–2016). Four publications were included from the first 5 years (2002–2006), six from the second (2007–2011) and five from the third 4-year period (2012–15).

### Country of origin, population and healthcare setting

Seven publications were from the US (one of these compared results with Australian hospitals); four were from Australia; and four from NZ, and one was NZ/Australian. No studies were from Canada. All but one of the US publications focussed on ethnic and racial minority clients other than Indigenous peoples, or diversity in general; one on American Indian and Alaskan native clients. The Australian and NZ publications all focussed on Indigenous clients. The healthcare settings that were the focus on the cultural competence intervention/measure were hospitals (*n* = 5 publications), primary healthcare services (*n* = 4) and specialist mental health (*n* = 4), antenatal (*n* = 1) and disability (*n* = 1) services.

### Intervention details

Although expressed using diverse terms (e.g. cultural sensitivity, cultural respect, diversity management), the aim of all 15 papers was to increase cultural competency through systems level-approaches. A detailed overview of intervention components is provided in Table 2. The symbol ✔ denotes evidence that the author(s) explicitly advanced adoption or support of the element of cultural competence, ~ denotes an implicit or inferred reference consistent with the intent of that element; and ✗ denotes no evidence for that element.

**Table 2:** Characteristics of the systems level interventions and measures to improve cultural competence

Publication	Aim	Implementation			Strategies										Outcomes							
First Author Year	Increased cultural competency	User engagement	Organisational readiness/commitment	Multiple sites of delivery	Audit and quality improvement		Organisation-level policies or strategies								Organisational systems			Client/practitioner encounter			Health outcomes	National outcomes
					Develop resources, tools and guidelines	Implementation of audit and monitoring	Cultural protocol or policy	Workforce diversity/ communication	Workforce CC training	Tailored services/programs	Org. environment: support, access, resource	Advocacy for cultural, economic & social	Promoted national CC standards implementn	Increase quality/access/participation	Improved resources/tools for providing cc	Identification of needs for improvement	Healthcare outcomes	Cultural respect/ communication	Client/family satisfaction	Practitione outcomes/satisfaction	Health outcomes or reduced disparity	Informed national standards
Chong in 2011 [36]	✓	✓	✓	✓	✓	✓	✓	✓	✓	✓	✓	✗	✓	✗	✓	✓	✗	✓	✗	~	✗	✓
Freeman 2014 [37]	✓	✓	✓	✓	✗	✗	✓	✓	✗	✓	✓	✓	✗	✗	✗	✓	✗	✓	✓	~	✗	✗
Liaw 2015 [38]	✓	✓	~	✓	✓	✓	✗	✓	✓	✓	✓	✗	✗	✗	✓	✓	✓	✓	✗	✗	✗	✗
Lieu 2004 [43]	✓	✗	✓	✓	✗	✗	✓	✓	✓	✗	✓	✗	✓	✓	✗	✓	✓	~	✓	✗	✓	✗
Noe 2014 [42]	✓	✗	✓	✓	✗	✗	✗	✗	~	✓	~	✗	✗	~	~	✓	✗	~	✗	~	✗	✗
O’Brien 2004 [39]	✓	✓	~	✓	✓	✓	✓	✓	~	✓	~	~	✓	✓	✓	✓	✓	~	✓	~	✗	~
O’Brien 2007 [40]	✓	✓	~	✓	✓	✓	✓	~	~	✓	~	~	✓	✓	✓	✓	✓	~	✓	✓	✗	~
Reibel 2010 [41]	✓	✓	~	✓	✓	✓	✓	✓	✗	✓	✓	✗	✗	✓	✓	✓	~	✓	~	✗	✗	✗
Weech-Maldonado 2012b [13]	✓	✗	✓	✓	✗	✗	~	✓	✓	✓	✓	✗	✓	✓	✓	✓	✓	✓	✓	✗	✗	✗
Whelan 2008 [44]	✓	✗	✓	✓	✗	✗	✓	✓	✗	✗	✓	✗	✗	✗	✓	✓	✗	✗	✗	✗	✗	✗
Whitman 2008 [45]	✓	✗	✓	✓	✗	✗	✓	~	~	✗	~	✗	✓	✓	✗	✓	✗	✓	✗	✗	✗	✗
Wiley 2009 [18]	✓	✓		✓	✗	✗	✓	✓	✓	✓	✓	✓	✓	✓	✗	✓	✗	✓	✓	✓	✗	~
O’Brien 2003 [46]	✓	~	~	✓	✓	✓	✓	~	~	✓	✓	~	✓	✓	✓	✓	✗	~	✗	✗		✗
Siegal 2003 [47]	✓	✓	~	✓	✓	✗	✓	✓	✓	✓	✓	✗	✓	✓	✓	~	✗	~	✗	✗		✗
Weech-Maldonado 2012c [48]	✓	✓	✓	✓	✓	✗	✓	✓	✓	✓	✓	✗	✓	✓	✓	~	✗	~	✗	✗		✗

#### Principles for implementation

The three most commonly reported principles for implementing systems-level interventions and measures to improve culture competence were: user engagement (*n* = 8), organisational readiness or commitment (*n* = 8) and delivery across multiple sites (*n* = 12). Other principles mentioned in publications included: being grounded in a social view of health, employing minority group staff, creating a welcoming service, supporting access, integrating cultural protocols, self-rating of services’ processes of change against the end goal of cultural security, using multi-level strategies and careful coordination.

## User engagement

Eight of the 12 papers described engagement and collaboration with affected population groups in the development and/or implementation of systems level cultural competence [18, 19, 36–41]. The frequency with which publications reported engagement with users in the development and delivery of effective cultural competence interventions indicates the importance of user involvement in identifying appropriate interventions. For example, Chong et al. [36] described a quality improvement framework designed collaboratively with Aboriginal Australians, and noted that hospitals with improved cultural sensitivity were those who engaged and had relationships with local Aboriginal Australian communities and commitment to supporting their Aboriginal workforce. This required senior management to prioritise and support this work and ensure that Aboriginal staff were trained to facilitate the process. Siegal et al. [19] identified the importance of users’ knowledge of cultural needs as one of 12 domains of US gold standard performance indicators that could be integrated within mental health services to measure the integration of cultural competence into daily operations. The emphasis on user involvement in part, was related to a recognition that healthcare users from diverse ethnic and racial backgrounds often have different worldviews to those underpinning the services of healthcare organisations and practitioners. For example, Wiley [18] noted conflict between worldviews of Maori disability clients which were based on Maori beliefs and traditions, compared with those of the mainstream services which were perceived by clients to fail to listen to the client or family. The consequence of not involving users was described by Wiley [18] whose evaluation of NZ’s national disability strategy found that Maori disability clients deemed the strategy to be less than optimally effective because it was adapted from mainstream to the Maori context rather than user-developed.

## Organisational readiness and commitment

The issue of organisational readiness for implementing cultural competence strategies was addressed in eight publications. For example, from the US, an exploratory study by Noe [42] focussed on the issue of the organisational readiness and capacity of 27 healthcare services of the Department of Veterans Affairs to adopt and implement native-specific services for American Indian and Alaska Native (AI/AN) veterans. They used an adapted Organisational Readiness to Change Assessment survey and profiled the availability of AI/AN veteran programs and interest in and resources for such programs. Other publications considered the commitment of managers to supporting cultural competence as one key enabler of implementation [5, 36, 37, 43–45].

## Multiple sites of delivery

All of the included publications considered the implementation of cultural competence across multiple, rather than single healthcare sites. The number of sites ranged from two primary healthcare services [37] to 66 hospitals [5].

### Strategies of systems-level interventions

The 12 systems-level intervention studies to improve cultural competence could be categorised into two broad types of approach: 1) audit and quality improvement approaches conducted across or within health services; and 2) evaluations of organisation-level systemic policies or strategies for cultural competence.

## Audit and quality improvement approaches conducted across or within health services

We found five intervention publications that reported on the trialling and/or implementation of audit and quality improvement approaches through targeted strategies [36, 38–41]. All five specifically focussed on Indigenous clients from Australia (3) and NZ (2) and were implemented within hospitals, primary healthcare services, mental health and antenatal services.

In these diverse healthcare settings, each study documented the development or tailoring of audit tools for the setting. In some studies, audit processes were used simply to identify the need for quality improvement. For example, persistent and significantly poorer Aboriginal perinatal outcomes motivated Reibel and Walker [41] to audit the usage frequency and characteristics of cultural responsiveness of maternal and child health antenatal services used by Aboriginal women in Western Australia. The utility of such studies lay in their identification of the extent of need for quality improvement. Other studies developed audit tools and tested them in trial sites. From Australia, for example, a case study by Chong [36] evaluated the development and piloting of an evidence based quality improvement framework to improve cultural sensitivity as it relates to Aboriginal health service delivery in five hospitals.

One study documenting the full audit and quality improvement cycle from NZ described the development and use of culturally and clinically reliable bicultural audit tools, the 25-item Consumer Notes Clinical Indicators (CNCI), to measure the achievement of culturally competent mental health nursing practice standards against standards of expected health care [39, 46].

Client ethnicity data was collected and linked to these quality measures. Another pre-post mixed methods study by Liaw [38] also documented the full audit and quality improvement cycle with 10 general practices. The study assessed the identification of Aboriginal clients, completion of health checks and management of chronic disease risk factors, and training and mentorship of staff to embed cultural respect in practice [38]. Monitoring of the frequency and characteristics of expected healthcare and client usage was then conducted [38, 39].

## Evaluations of service-level policies or strategies for cultural competence

We found six evaluations within or across service-level policies or strategies for cultural competence [5, 18, 37, 43–45]. Four were from the US, one compared US and Australian hospitals, one was from Australia and one from NZ. The evaluations of service-level policies and strategies for cultural competence considered very diverse populations and healthcare settings. One study evaluated the effects of organisational cultural competence policies on healthcare and health outcomes [43]. This US study by Lieu [43] examined the cultural and linguistic competence policies of five health plans in three states, and their association with quality of managed care for Medicaid-insured children of non-English speakers with asthma.

Two studies evaluated the effect of cultural competence on clients’ experiences of care. Weech- Maldonado et al. [5] explored whether greater cultural competence in hospitals improved client experiences, particularly for ethnic/racial minority clients, by correlating scores from the US national Consumer Assessment of Healthcare Providers and Systems Hospital Survey with those from the Cultural Competence Assessment Tool for Hospitals. Freeman et al. [37] identified cultural respect strategies in two primary healthcare case studies. The strategies were: being grounded in a social view of health, including advocacy and addressing social determinants; employing Aboriginal staff; creating a welcoming service; supporting access through transport, outreach, and walk-in centres; and integrating cultural protocol. They also identified client experiences and barriers to cultural respect (communication difficulties; racism and discrimination; and externally developed programs).

Three studies evaluated the extent to which (or how well) organisational or national cultural competence policies/strategies had been implemented. Whitman and Davis [45] considered whether the policies and practices used by Alabama hospitals met the national US National CLAS standards. Whelan et al. [44], compared diversity management strategies by senior hospital managers in Pennsylvania with those of Sydney hospitals to determine how well they implemented best practice diversity management. The diversity management activities evaluated included planning, stakeholder satisfaction, diversity training, human resources, health care delivery, organisational change, diversity performance, and external and internal influences on racial/ethnic diversity initiatives. From NZ, Wiley [18] suggested a need for improved coordination, collaboration, workforce development, information and resources, and community engagement in the implementation of the NZ Disability Strategy.

### Outcomes of systems-level cultural competence interventions

Four types of outcomes of systems-level cultural competence were identified. These were: 1) organisational systems outcomes including improved resources/tools for providing cultural competence and identification of needs for improvement; 2) outcomes related to the client/practitioner encounter including identification of cultural respect/communication, client/family satisfaction, and practitioner outcomes/satisfaction; 3) healthcare and health outcomes; and 4) broader outcomes such as informing national standards for cultural competence.

#### Organisational healthcare systems outcomes

Seven of the 12 intervention papers described outcomes of improved resources/tools for providing cultural competence, and all 12 papers identified needs for systems improvements in promoting cultural competence. The publications that reported audit and quality improvement approaches [37–41] considered these approaches to be relevant for establishing benchmarks for health service utilisation and quality and to driving system-wide healthcare action against national standards, and reported improved healthcare outcomes. Audits provided a quality mechanism for identifying aspects of health care where improvements in cultural competence were needed [39, 40] and a commitment by healthcare administrators to achieving culturally-competent policy, health service delivery and environments [36]. Liaw et al. [38] found encouraging improvements in primary healthcare staff members’ scores on cultural competency scales, and that audits, training and mentoring led to increases in Aboriginal health checks and improved management of clinical risk factors.

All 12 publications identified areas of further need for improved implementation of systems approaches to cultural competence. For example, Reibel and Walker [41] identified that only nine of the 42 Western Australian antenatal services which reported use by Aboriginal women, had provided both culturally secure and consistent antenatal care. Few services incorporated Aboriginal specific antenatal protocols/program, employed Aboriginal Health Workers, or were accessed regularly by Aboriginal women. The authors suggested that the cultural responsiveness indicators used in the audit established benchmarks as a starting point for future service delivery improvement [41]. Freeman et al. [37] concluded that service-level strategies were necessary to achieving cultural respect and had the potential to improve Aboriginal and Torres Strait Islander health and wellbeing.

Similarly, studies based on surveys of healthcare system administrators also identified needs for systems improvements in promoting cultural competence. For example, Noe et al. [42] determined that program needs, leaders’ practices and communication predicted the provision of care that staff considered met the needs of AI/AN veterans, but not implementation of native-specific services. Assessment of organisational readiness could assist in developing strategies for adopting and implementing native-specific programs and services. At a broader scale, Whelan et al. [44]’s comparison of Australian and US hospitals found that both systems can do much more to implement best practices in diversity management. Australian hospitals scored higher on organisational change indicators; US hospitals on human resource indicators, but there was more similarity than difference. They concluded that despite 30–40 years of “multicultural health”, hospitals in neither country has achieved best practice. Similarly, the study by Whitman and Davis [45] of Alabama hospitals found that although these hospitals were taking initial steps to prepare for a diversifying client population, only 13% hospitals met all four of the linguistic CLAS standards, and 19% met none. That is, enforcement of national legislation was inconsistent and legislation in itself does not necessarily guarantee health service implementation.

#### Client/practitioner encounter outcomes

Study outcomes also included the increased involvement of clients and their families in their own healthcare, improved relationships in the client/practitioner encounter, and consequently increased health service access and frequency of visits. For example, Weech-Maldonado et al. [5] found that greater cultural competence was positively associated with some measures of clients’ experiences with care (doctor communication, overall hospital rating and hospital recommendation). There were greater relative benefits for non-English-speaking non-Hispanic whites. Freeman [37] found that 22 staff and 21 clients reported positive appraisals of the achievement of cultural respect. While not significant, Reibel and Walker [41] found that Aboriginal women increased utilisation to the nine culturally responsive antenatal services from 3 to 5 visits.

#### Health outcomes

Health outcomes were also reported. Lieu et al. [43] found that Medicaid-insured children of non-English speakers with asthma clients of managed care practice sites with the highest cultural competence scores were less likely to be underusing preventive asthma medications based on parent report at follow-up (odds ratio: 0.15; 95% confidence interval: 0.06–0.41 for the highest vs lowest categories) and had better parent ratings of care. O’Brien et al. [40] found that implementation of their NZ audit and quality improvement approach in mental health services enabled measured improvements in clients’ and families’ involvement in health care and ultimately improved recovery. Thus, cultural competence improved the quality of healthcare and produced health outcomes.

#### National outcomes

Finally, there were national policy outcomes from cultural competence interventions. For example, elements of the quality improvement toolkit developed for hospitals by Chong et al. [36] were included in the Australian Council of Healthcare Standards; this provided a further driver for change. Wiley [18] demonstrated that there was commitment to achieving a culturally-competent NZ national disability strategy, health service delivery and workplace environment to benefit Maori people with disabilities. The implementation of the strategy required collaboration across sectors, accountability structures and effective evaluation tools, as well as collaboration between Maori people with disabilities and their families, and the disability sector. As stated by Wiley [18], these “provide cautionary lessons that Indigenous [and other ethnic and racial] peoples and governments in other countries can use in the development of culturally comprehensive… policy.”

### How has cultural competence at a systems-level been measured?

Many of the intervention studies incorporated measurement instruments, but we also found three studies that specifically reported the development of quality improvement and other indicators to measure cultural competence at systems levels. Of these, two were from the US and focussed on diverse ethnic and racial groups, and one from NZ which described the development of clinical and cultural competence indicators for mental health service improvements for Maori clients. The rigorous processes of indicator development and testing described demonstrated the considerable resources which have been invested into developing and piloting instruments to audit service performance across sectors within health [19, 46].

A US study by Siegal et al. [47] described the development of gold standard indicators that could be integrated within mental health services to measure the integration of cultural competence into daily operations. The US Substance Abuse and Mental Health Services Administration Centre developed, pilot tested and established the psychometric properties of the measures using an expert panel to rate the measures according to their importance, feasibility, reliability and likely stage of implementation. The result was a checklist of 85 performance measures, clustered within 12 domains: commitment of the organisation to cultural competence; integration of cultural competence within organisation; activities related to cultural competence in organisational components; cultural competence advisory committee; knowledge of cultural needs of target population; knowledge of cultural needs of users; linguistic capacity; services; cultural competence training and education; recruitment, hiring and retention; outcomes; and consumer and family education.

Also targeting mental health services, O’Brien et al. [46] described the development and validation of the NZ culturally and clinically reliable bicultural audit tools to measure the achievement of their mental health nursing practice standards (evaluation of their implementation is described above). The CNCI audit tool was based on identification of ‘critical events’ from nursing notes in consumer’s case notes. Critical events were ‘non-sentinel rate- based clinical indicators considered crucial to achievement of practice standards which if not achieved, identified a need for immediate rectification [46]. Of 100 clinical indicator statements, 25 valid and reliable indicators were considered to be crucial to the achievement of the NZ standards. The measures were also considered to be relevant to mental health nursing internationally by providing a framework for improving practice against standards of expected health care [40].

Weech-Maldonado et al. [48] described the development of the Cultural Competency Assessment Tool for Hospitals (CCATH) to reflect the six US National Quality Forum domains and 14 CLAS standards. An initial draft of the tool was then pilot tested to ensure ease of administration, comprehensibility and clarity, and to minimise response burden. It was revised, then field tested with five Californian and Pennsylvanian hospitals. The pilot testing resulted in the redesign and reduction of the survey to 28 items based on four overarching domains: culturally competent care; human resource management; translation and interpretation; and leadership, climate and strategies.. The 28-item version was then focus tested with hospital staff from seven US states and interviews with hospital administrators. Final revisions were then completed. The study found that the CCATH scales were reliable, and that the CCATH can be used to evaluate hospital performance in cultural competency and identify improvements. Not for profit hospitals had higher CCATH scores than for profit hospitals.

### The quality of available evidence

Only one of the 12 intervention studies was rated of strong quality [43]. Four studies were rated of moderate quality, and seven of weak quality, with lack of consistently strong methodology across the majority of assessed criteria. The quality of the three measurement studies was not assessed.

There were no randomised controlled studies. One was a prospective cohort study, which used multiple data sources [43]; one a pre-post intervention cohort analytic study [39]. Six provided evidence from controlled single timepoint audits or measures of cultural competence across multiple healthcare services [5, 39–42, 44, 45]. The remaining three studies used an exploratory qualitative case study design [18, 36, 37].

### Limitations

The publications reviewed were identified using a rigorous search strategy which incorporated electronic databases, websites/clearinghouses and reference lists of reviews designed to discover peer and non-peer reviewed publications. Therefore, it is highly likely that the studies in this review are representative of published cultural competence research from the US, Canada, NZ and Australia. However, being a non-exhaustive search strategy, it is possible some relevant publications were not found. Additionally, due to the breadth and complexity of systems-level cultural competency, this review only included studies which explicitly aimed to improve, or included an indicator of cultural competency, possibly excluding studies which implicitly aimed to increase cultural competence. Given the complexity of systems-level approaches, studies may have used terms (such as diversity management, community engagement, or quality improvement for particular health issues and population groups) which were not included in our search. To further develop the evidence base on systems-based interventions to improve cultural competency and their impacts on relevant outcomes, it is important that studies use consistent terminology and explicitly address this in their aims.

## Discussion

**Fig. 3:**
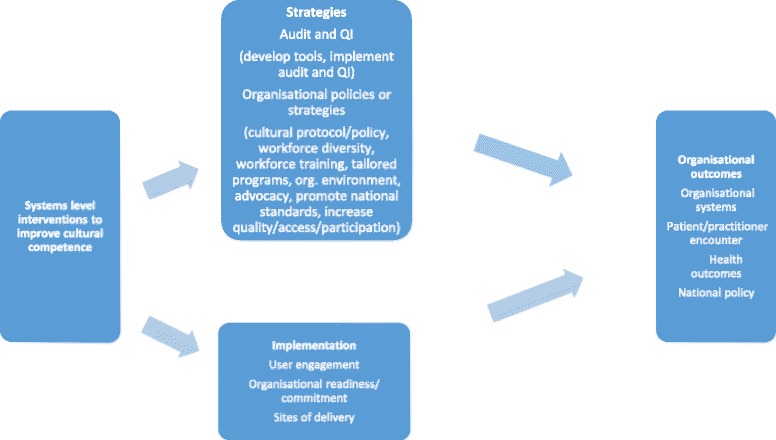
The implementation principles, strategies and outcomes of systems approaches to cultural competence

Systems approaches focus attention on how things work together to achieve an intended outcome and on understanding of the ‘whole’ system [11]. By understanding how things are connected to each other within a whole entity, systems can be changed to produce better outcomes [11]. Derived from the thematic analysis of the interrelated systems implementation principles, strategies, and outcomes identified in this systematic scoping review, Fig. 3 below provides a preliminary generic framework for systems-level approaches to cultural competence; however the framework would require tailoring for specific country/setting/populations and types of health care services provided.

All of the 12 intervention studies explicitly iterated some core principles for implementing cultural competence across healthcare systems. There was variation across studies in the explication of important implementation principles. The three highlighted in this review were user engagement in the development and/or implementation of strategies, organisational readiness, and delivery across multiple sites. Other reviews of the cultural competence literature have also reported the value of user engagement to ensure congruence of strategies with the cultural beliefs, values and practices of the affected population groups [4, 23, 27, 28]. However, studies in this review provided innovative systematic ways to embed user engagement into healthcare. These included providing services that are based on the worldviews/paradigms and control by the user group (e.g. [18, 37]), audit indicators for user’s consent, choice, mutual goal setting and review; assessment by cultural advisors; specific cultural preferences and access to these; and support for access to traditional medicine/remedies (e.g. [19, 46]). The finding that some mainstream systems level interventions were less users (e.g. [18]), suggests that similar to other cultural competence strategies, achieving improvements in systems-level cultural competence approaches is dependent on early collaboration with affected user groups and networks with user-controlled health services.

The issue of organisational readiness/commitment has also been identified in other reviews. It makes sense that organisational commitment is required for systems approaches, given the complexity required to coordinate organisational sub-systems to work together in a coordinated way to achieve culturally competent healthcare provision [49]. Organisational commitment is also required because cultural competence is just one of many investment priorities facing healthcare organisations, and as [50] argued, the available financial incentives for cultural competence remain “not always clear or consistent”. Studies have suggested that the business potential provided by quality culturally competent care should be recognised in national cultural competence policies or strategies by linking these with quality care incentive payments [13, 50]. However, this systematic search found no intervention studies of the impact of financial incentives or the cost effectiveness of systems-level approaches; hence it remains unclear whether systems-level cultural competence is a cost-effective strategy.

An interesting review finding was that all intervention studies of systems approaches were implemented across multiple sites. This may be a result of efforts to scale up interventions and for maximum reach and outcomes, or simply due to a quest for stronger research outcomes. Further research is needed within singular health organisation and across multiple organisations.

The two key types of intervention strategies to embed cultural competence within health systems identified were: audit and quality improvement approaches; and service-level policies or strategies for cultural competence. Audit and quality improvement approaches were implemented across diverse healthcare settings and relevant to improving healthcare practice against national benchmark standards. They resulted in improved relationships with local communities, increased health service access and frequency of visits, and the increased involvement of clients and their families in their own healthcare and ultimately improved recovery following mental illness [38, 40, 41]. Evaluations of service-level policies and strategies for cultural competence included cultural protocols or policies such as for interpretor services and translation of materials; workforce diversity and training; the tailoring of services or programs; providing a conducive organisational environment; advocacy; promoting national standards; and increasing access, participation and quality. Studies found that compliance with service level policies resulted in improved client and family satisfaction and health outcomes such as improved compliance with medication [5, 43]. These findings were extended by a promising recent paper, published post- search, which found that a systemic, multifaceted and organisational level cultural competency initiative in two hospitals led to overall performance improvement, and outperforming of control hospitals with respect to diversity climate [49].

We could not determine the overall effectiveness of systems-level interventions to reform health systems because interventions were context specific to both the country, setting and population, and to the type of health care services concerned. As well, there were either too few comparative studies, or studies did not examine the same outcome measures. The preponderance of the literature about systems-level cultural competence interventions focussed on qualitative process evaluations, which explore the concepts and issues and described the interventions and formative or intermediate outcomes. It is likely that this is because the field is still in the relatively early stages of development, therefore there has not been enough elapsed time for follow-up studies and thus we do not know the full impact of systems-level cultural competence interventions on healthcare services or their clients. Further, almost every included study utilised a different measure, suggesting that measures of cultural competence at systems level require further elucidation. The domains of the mental health performance measures for administrative and service entities [19] and more recent CCATH for application in hospitals [48] suggest that important outcome measures are: the cultural competence of clinical/health care (including consumer representation and care delivery), human resource management (including workforce diversity and training), translation and interpretation services, and organisational commitment, leadership and data management and quality improvement systems. The findings of this review suggested that also useful are measures of the health outcomes from interventions and broader research translation to effect national or jurisdictional policies related to cultural competence in healthcare. Documented measures (e.g. [46–48]) are currently based on the perceptions of healthcare managers/administrators who are likely to have the required information to complete them [49]; however, given the importance of user engagement, there is a strong case for incorporation of patient perspectives in evaluating the cultural competence of healthcare interventions. In the case of national level policy interventions, the same would apply to the inclusion of policy makers and public perspectives. The effectiveness of an intervention would be evaluated based on improvement in outcome measures. While tailoring across healthcare setting is necessary, as suggested by Brach and Fraser [50], the consistent use and reporting of systems-level cultural competence measures within each setting type would provide an important tool for comparable quality improvement efforts to build a strong evidence base.

Identified gaps in the literature included a need for cost-effectiveness studies of systems approaches to improve cultural competence, further explication of the effects of cultural competence on client experience, and studies to further explore the ultimate effect of cultural competence on improving health outcomes and reducing ethnic and racially-based healthcare disparities. Doing so will require a concerted commitment to adequately funding the implementation and monitoring of such initiatives [50, 51].

### Implications

Few studies have previously examined the impact of systems-level approaches to cultural competence [22, 25, 49]. While substantial evidence suggests that systems-level cultural competence should work, our finding of only 12 intervention studies means that we cannot confidently determine the extent to which systematic approaches to cultural competence are useful for improving clients’ experiences of healthcare and their health outcomes. Rather, there is little guidance for healthcare organisations about how to identify what mix of cultural competence strategies works in practice; when and how to implement them properly [22], or whether their investment in cultural competence interventions will have the intended effects on client experiences or health outcomes.

## Abbreviations

ACP Journal club: American College of Physicians Journal Club
AI / AN: American Indian and Alaska Native
AIATSIS: Australian Institute of Aboriginal and Torres Strait Islander Studies
ApaI-ATSIS: Australian Public Affairs Information Service – Aboriginal and Torres Strait Islander Studies
ATSIHealth: Aboriginal Torres Strait Islander Health
Sahps: Consumer Assessment of Healthcare Providers and Systems survey
CANZUS nations: Canada, Australia, New Zealand and the United States NZ: New Zealand
CASP: Critical Appraisal Skills Programme quality assessment tool
Step: Cultural Competence Assessment Tool for Hospitals
CINAHL: Cumulative Index to Nursing and Allied Health Literature
CLAS: Culturally and Linguistically Appropriate Services
CNCI: Consumer Notes Clinical Indicators
Cochrane DSR: Cochrane Database of Systematic Reviews
CQI: Continuous quality improvement
DARE: Database of Abstracts of Reviews of Effects
EBM Reviews: Evidence Based Medicine reviews
Afpp: Effective Public Health Practice Project quality assessment tool
FAMILY-ATSIS: Family Aboriginal Torres Strait Islander Studies
PARENTS: Primary Application Information Service
PPAQ: Professional Practice Audit Questionnaire
PRISM: Preferred Reporting Items for Systematic Reviews and Meta-Analyses
US: United States
